# Inhibition of Seed Germination in *Portulaca oleracea* L. by *Rhus typhina* L. Extract Is Mediated via Suppression Activities of Amylase Rather than Antioxidative Enzymes

**DOI:** 10.3390/plants15152310

**Published:** 2026-07-28

**Authors:** Yingmei Ma, Jiaqi Feng, Tergun Bau, Xinghua Zhao, Feng Han

**Affiliations:** 1 College of Desert Control Science and Engineering, Inner Mongolia Agricultural University, Hohhot 010018 , China; fengjq357@126.com (J.F.); zhaoxinghua2007@126.com (X.Z.); 2 State Key Laboratory of Water Engineering Ecology and Environment in Arid Area, Inner Mongolia Agricultural University, Hohhot 010018, China; 3 Research Center for Agricultural, Forestry, and Fishery Sciences, Inner Mongolia Agricultural University, Hohhot 010018, China; truun2010@126.com; 4 College of Grassland Sciences, Inner Mongolia Agricultural University, Hohhot 010018, China; hfnmd@imau.edu.cn

**Keywords:** *Portulaca oleracea* L., *Rhus typhina* L., weed control, antioxidative enzyme activity, amylase activity

## Abstract

*Rhus typhina* L. is widely recognized as a landscape ornamental plant and its extracts have demonstrated biological activity against multiple weed types. This makes it a promising candidate in sustainable agriculture as a bioherbicide by offering a solution to mitigate the growing problem of herbicide resistance. Therefore, this study was designed in agar culture to analyze the effect of powders of the branch, fallen leaf, and pericarp from *Rhus typhina* L. against seed germination of *Portulaca oleracea* L. and evaluate its inhibitory mechanisms based on anatomical structure and the activities of antioxidative enzymes, α-amylase, and β-amylase. The main allelopathic compounds were also identified based on high-performance liquid chromatography–mass spectrometry (LC-MS), and their bioherbicidal effects were evaluated. *Brassica napus* L. was used as the object for biosafety evaluation. The results showed that the seed germination of *Portulaca oleracea* L. under treatment with powder extract from *Rhus typhina* was inhibited by exhhibting swelling and deformation in endosperm cells and chaotically scattered starch granules in seed embryos with a shorter radicle length than the control. During the period of treatment, antioxidative enzymes were quickly activated to scavenge the suddenly surging reactive oxygen species (ROS) as a measure of boosted malonaldehyde (MDA) content. Antioxidative enzyme (POD, SOD, and CAT) activities were also activated to mitigate the oxidative damage. In contrast, both α-amylase and β-amylase activities were significantly suppressed. A total of 16 allelochemicals were identified in the branch, fallen leaf, and pericarp of *Rhus typhina* L. Among these, gallic acid, methyl gallate, and tyrosol were identified as exhibiting the most significant inhibitory effect during the whole germination process of *Portulaca oleracea* L., resulting in browned seedlings and stunted radicle length compared with the control, while far weaker inhibitory effects were found against *Brassica napus* L. In summary, extracts from *Rhus typhina* L. targeted α-amylase and β-amylase activities rather than antioxidative enzyme activities to inhibit the germination of *Portulaca oleracea* L. Gallic acid, methyl gallate, and tyrosol were the key compounds that inhibited its germination.

## 1. Introduction

Canola (*Brassica napus* L.) is an oil-rich crop cultivated as a significant source of vegetable oil production globally. It is also a significant crop in Canada in agricultural sector by producing approximately 20 million tons and contributing 25% of the country’s agrarian income annually [1]. In the United States, since 1991, its yield has grown to more than 2 million acres per year. Yet, growth in production has not kept up with demand, and 69% of canola oil is still imported for domestic consumption [2]. However, canola production and its yield are significantly hindered by the influence of weeds. Its yield per hectare could be reduced by 20% due to the invasion of weeds [3]. Weeds such as annual ryegrass (*Lolium rigidum* Gaudin), wild oat (*Avena fatua* L.), and wild radish (*Raphanus raphanistrum* L.) are responsible for the highest yield losses for canola crops in Western Australia [4]. Specifically, *Portulaca oleracea* L., commonly known as purslane, is a worldwide weed species belonging to the family Portulacaceae and has been called a “Global Panacea”, which could cover ground quickly by expanding its aboveground canopy at the seedling stage of canola [5]. Moreover, its seed production is significantly high and can be spread to the crop field by wind, water, and animals, resulting in high weed seed banks in the soil and high germination [6,7]. It can also tolerate poor soil conditions and cold temperature, which leads to its higher survival than other plant species [8]. In the short term or under poor soil conditions, it can take over the crop field area very easily at the vegetative stage of canola growth [9]. Therefore, control of *Portulaca oleracea* L. plays a significant role in the security of canola production in the field.

Currently, there are varieties of chemical herbicides used to control *Portulaca oleracea* L. In the United States and other countries, herbicides constitute two thirds of total chemical applications in agriculture fields [10] to control *Portulaca oleracea* L. effectively [11]. However, long-term usage of chemical herbicides has led to weed tolerance to some herbicide types [12]. Studies have shown that glyphosate, a common herbicide used since 1974, has carcinogenic toxicities for human beings [13] and leads to herbicide resistance to weeds [14]. Related research also demonstrated that 2,4-D has a significant impact on soil microbial activities and this impact is modulated by herbicide application rates and soil types [15]. Moreover, crop field environmental pollution is also appearing due to the usage of herbicides [16]. Therefore, developing an effective bioherbicide that is environmentally friendly could provide a solution to these problems. Previously, the authors identified allelopathic effects from water extract and powder of *Rhus typhina* L. which could effectively inhibit the seed germination of *Portulaca oleracea* L., *Chloris virgata* P.Durand, and *Convolvulus arvensis* while showing non-significant inhibition of the seed germination of canola [17,18]. Additionally, extracts of *Rhus typhina* L. root extracts were shown to be capable of effectively inhibiting the growth of *Tagetes erecta* L. and soil microbial activity [19]. *Castanea crenata* Siebold & Zucc. leaves also showed strong inhibition against the germination and seedling growth of barnyardgrass (*Echinochloa colona* (L.) Link), lettuce (*Lactuca sativa* L.), and radish (*Raphanus sativus* L.), appearing as a promising material for managing weeds, especially against dicot weeds [20]. Therefore, usage of this allelopathic effect from *Rhus typhina* L. could be a sustainable weed management approach in the crop field.

The seed is the basis and the most important tissue for *Portulaca oleracea* L. in maintaining its successful reproduction and further invasion as a weed [21]. Therefore, the germination process of *Portulaca oleracea* L. was use in this studyas it could provide insights that may help to identify the mode of action of bioherbicides and their successful product development. Seed imbibition leads to a significant increase in reactive oxygen species (ROS) content mainly due to the activation of respiration and metabolic recovery during the seed germination process. Enzymes like superoxide dismutase (SOD), catalase (CAT), glutathione peroxidase (GPX), and ascorbate peroxidase (APX) [22] are produced to reduce the damage caused by excessive ROS, including hydrogen peroxide (H_2_O_2_), superoxide ion (O_2_•^−^) [23], singlet oxygen (^1^O_2_), and hydroxyl radical (•OH) [24], which form relatively stable, long-lasting and permeable H_2_O_2_ [25] and regulate its germination processes [25]. During the early stage of seed germination, SOD is used to deactivate and catalyze O_2_•^−^ to H_2_O and O_2_ to reduce harmful effects on cell structures. In germinated seeds, CAT thus specifically responds to excessive H_2_O_2_ that is harmful to fatty acid β-oxidation [26]. Ascorbate peroxidase (APX) could also catalyze the oxidation of ascorbate to monodehydroascorbate and then to dihydroartemisinin (DHA) [27]. For instance, seed treatments (CaCl_2_) significantly improved germination with decreased content of MDA, hydrogen peroxideH_2_O_2_ and SODactivity in safflower (*Carthamus tinctorius* L.) [28]. However, peroxide activities in purple nutsedge (*Cyperus rotundus* L.) decreased under 40% of wedelia (*Wedelia trilobata* (L.) Hitchc.) leaf extract treatment indicating the failure of the plant’s antioxidant defense to inhibit weed growth [29].

Amylase also facilitates seed germination by hydrolyzing starch into sugar [30]. For instance, during wheat (*Triticum aestivum* L.) germination, starch is hydrolyzed by free β-amylase in the first 24 h [31]. In the subsequent 24 h, α-amylase and β-amylase work synergistically to hydrolyze more starch, while in later stages of seed germination, α-amylase becomes the predominant active form and β-amylase gradually diminishes [31]. In a study tracking 0–8 days of soaking rice (*Oryza sativa* L.), wheat, ragi (*Eleusine coracana* (L.) Gaertn.) and bajra (*Cenchrus americanus* (L.) Morrone), α-amylase, β-amylase, and protease activities increased significantly and maximum α-amylase was observed on day 6, β-amylase during days 4–6, and proteases on day 2 [32]. Therefore, in this study, the activities of these enzymes were the subject of evaluation to indicate the effect of powder extract against seed germination of *Portulaca oleracea* L.

Paraffin embedding is applicable to various types of tissues to give tissues a uniform stiffness that is needed for sectioning within a microtome. Examples include dissecting the rice (*Oryza sativa* L.) shoot apex and immature inflorescence [33]. By using this method, a considerable number of holes and cracks were identified in the outer and inner endosperm cells during seed dormancy under treatments of H_2_SO_4_ and gibberellin [34]. Thus, this method was employed to reveal the dynamic structural changes during seed germination of *Portulaca oleracea* L. under treatment of *Rhus typhina* L. extract. In addition, allelopathic chemical compounds play an important role in inhibiting seed germination. By GC-MS and HPLC analyses, gallic, protocatechuic, p-hydroxybenzoic, caffeic, ferulic, ellagic, and cinnamic acids were identified and quantified from *Castanea crenata* Siebold & Zucc. leaf, of which ellagic acid was in the highest quantity (2.36 mg/g DW). Further purification via column chromatography combined with spectral characterization using H-1- and C-13-NMR, IR, and LC-MS resulted in purified compound as 2α, 3β, 7β, 23-tetrahydroxyurs-12-ene-28-oic acid from the methanolic leaf extract of *Castanea crenata* Siebold & Zucc.(0.93 mg/g DW) with potent inhibition against the growth of the weed *Echinochloa crus-galli* (L.) P. Beauv. in agricultural practice [20]. Hexane is another identified compound from the aerial parts of *Andropogon virginicus* L. by gas chromatography–mass spectrometry (GC-MS) which significantly inhibited the α-amylase activity of the weed [35].

Therefore, this study was designed to (1) structurally visualize the dynamic seed germination changes of *Portulaca oleracea* L. and *Brassica napus* L., (2) qualify the activities of antioxidative enzymes and amylase during the germination of *Portulaca oleracea* L., (3) compare the species variance between *Portulaca oleracea* L. and *Brassica napus* L. under treatment with powder extract from *Rhus typhina* L., (4) identify the constituent compounds from powder extract of *Rhus typhina* L. that inhibit the seed germination of *Portulaca oleracea* L., and (5) evaluate their bioherbicidal effect against the seed germination of *Portulaca oleracea* L.

## 2. Results

### 2.1. Content of Malondialdehyde During Germination of Portulaca oleracea *L.* and Brassica napus *L.*

**Figure 1:**
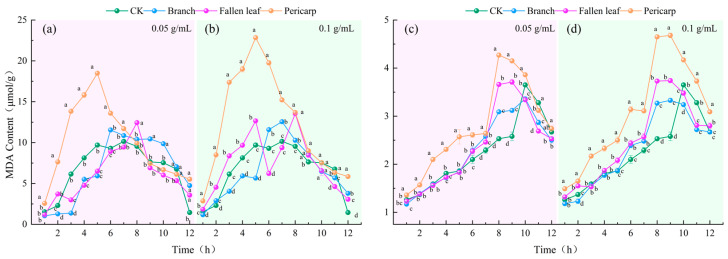
Malondialdehyde content during seed germination of *Portulaca oleracea* L. treated with powder of *Rhus typhina* L. with concentrations of 0.05 g/mL (**a**) and 0.1 g/mL (**b**) and *Brassica napus* L. treated with powder of *Rhus typhina* L. at concentrations of 0.05 g/mL (**c**) and 0.1 g/mL (**d**). Different letters above each data point indicate significant differences among different treatments at each time point at α ≤ 0.05.

During seed germination of purslane, both concentrations of pericarp and fallen leaf treatments induced the accumulation of malondialdehyde (MDA) content (Figure 1a,b). However, higher-concentration treatment induced higher production of MDA (22.69 μmol/g) than a lower concentration (18.44 μmol/g) and such a stress effect was significantly higher in the early stage (1–6 h) of seed germination than the later stage (7–12 h), which indicated a sharp burst of reactive oxygen species as MDA content. Under a lower concentration of treatment, only pericarp induced accumulation of MDA in the early stage of seed germination when compared with fallen leaf and branch treatments (Figure 1a), which demonstrates that pericarp exerts the highest inhibitory effect against the seed germination of purslane among the tested treatments.

During the seed germination of canola, the production trend of MDA content was similar to that of purslane. However, the peak level of MDA production (4.68 μmol/g) was 9 h after imbibition, which was later than 5 h after imbibition in purslane (Figure 1c,d). When comparing the peak values across the two species, the overall MDA content was lower in canola (4.52 μmol/g) than purslane (22.54 μmol/g; Figure 1c,d). Under a low concentration (0.05 g/mL) of treatment, only the pericarp of *Rhus typhina* L. had an effect on the induction of MDA compared to the control. Under a high concentration (0.1 g/mL), pericarp, fallen leaf, and branch induced significantly higher contents of MDA than the control, where non-significant differences were found between the fallen leaf and branch (Figure 1d).

### 2.2. Peroxidase Activity During Germination of Portulaca oleracea *L.* and Brassica napus *L.*

**Figure 2:**
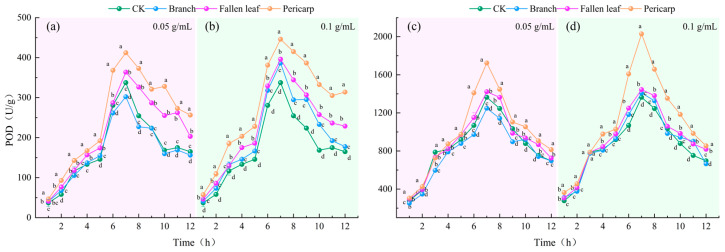
Peroxidase activity during seed germination of *Portulaca oleracea* L. treated with powder of *Rhus typhina* L. at concentrations of 0.05 g/mL (**a**) and 0.1 g/mL (**b**) and *Brassica napus* L. treated with powder of *Rhus typhina* L. at concentrations of 0.05 g/mL (**c**) and 0.1 g/mL (**d**). Different letters above each data point indicate significant differences among different treatments at each time point at α ≤ 0.05.

For purslane, peroxidase (POD) activities from all treatments were significantly higher than control with a similar dynamic trend under both concentrations. The general average POD activity in purslane (446.3 U/g) was significantly lower than in canola (2025.73 U/g) across all treatments with both concentrations (Figure 2a–d), which demonstrates that the higher maintained POD activities from canola compared to purslane mitigate oxidative damage more effectively.

Under treatments with pericarp, fallen leaf, and branch at both concentrations, the variance of POD activity in the early stage (1–7 h) was lower than in the later stage (7–12 h) of germination in purslane (Figure 2a,b), which indicated POD activities were strongly activated during the later stage of germination. Specifically, POD activity under treatment of pericarp (229.45 U/g) at a higher concentration (0.1 g/mL) was significantly higher than with fallen leaf (168.45 U/g) and branch (183.70 U/g) after 5 h of imbibition (Figure 2b), which indicated less inhibition effect on POD activity.

The dynamic change trend in POD activity during canola germination was similar to that in purslane, though its overall content was lower than in canola (Figure 2c,d), which indicated that the extract from *Rhus typhina* L. exerted a lower inhibitory effect on canola. Under pericarp treatment (1406.7 U/g) with a low concentration (0.05 g/mL) at the peak point, POD activity was significantly higher than in the control, fallen leaf, and branch (Figure 2c). Under a high concentration (0.1 g/mL) of pericarp, the induced POD activity (1605.5 U/g), fallen leaf (1253.71 U/g), and branch (1185.6 U/g) values were significantly higher than in the control (1065.5 U/g) after 6 h of imbibition (Figure 2d).

### 2.3. Superoxide Dismutase Activity During Germination of Portulaca oleracea *L.* and Brassica napus *L.*

**Figure 3:**
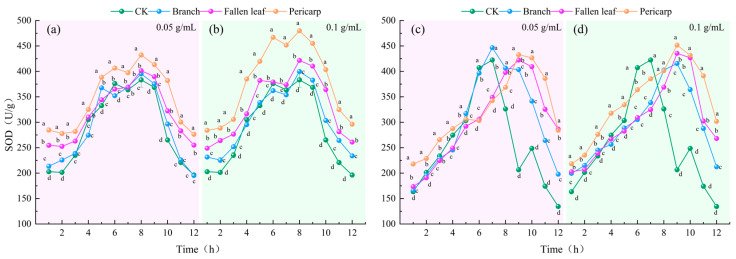
Superoxide dismutase activity during seed germination of *Portulaca oleracea* L. treated with powder of *Rhus typhina* L. at concentrations of 0.05 g/mL (**a**) and 0.1 g/mL (**b**) and *Brassica napus* L. treated with powder of *Rhus typhina* L. at concentrations of 0.05 g/mL (**c**) and 0.1 g/mL (**d**). Different letters above each data point indicate significant differences among different treatments at each time point at α ≤ 0.05.

The overall activity of superoxide dismutase (SOD) activity was similar with two peaks during the seed germination of both purslane and canola, which indicated their defensive effect due to treatments with extract from *Rhus typhina* L. (Figure 3a–d).

Under all treatments at both concentrations, the SOD activities during purslane seed germination were all boosted due to stress from the extract of *Rhus typhina* L. Specifically, under a high concentration (0.1 g/mL) of pericarp treatment, SOD activity (358.77 U/g) was significantly higher than that with fallen leaf (317.61 U/g), branch (295.76 U/g), and the control (295.76 U/g) after 4 h of imbibition (Figure 3b), which indicated that the extract from pericarp exerted a higher inhibitory effect than the other two parts.

During the seed germination of canola, the variance among all treatments of pericarp, branch, and fallen leaf at both concentrations was significantly higher than in the control, which may suggest that powder extract from *Rhus typhina* L. boosted high antioxidative capacity during the germination of canola (Figure 3c,d).

### 2.4. Catalase Activity During Germination of Portulaca oleracea *L.* and Brassica napus *L.*

**Figure 4:**
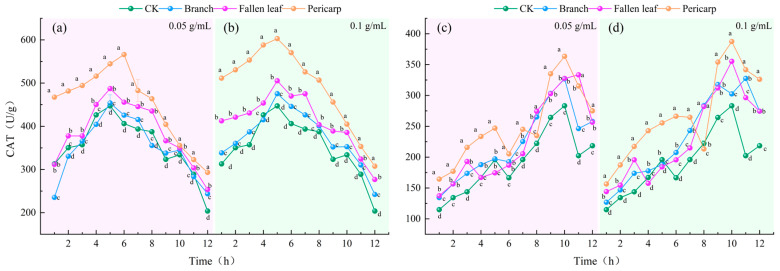
Catalase activity during seed germination of *Portulaca oleracea* L. treated with powder of *Rhus typhina* L. at concentrations of 0.05 g/mL (**a**) and 0.1 g/mL (**b**) and *Brassica napus* L. treated with powder of *Rhus typhina* L. at concentrations of 0.05 g/mL (**c**) and 0.1 g/mL (**d**). Different letters above each data point indicate significant differences among different treatments at each time point at α ≤ 0.05.

Treatment with extract of *Rhus typhina* L. significantly altered catalase (CAT) activity in both species. Across the entire germination process, the overall CAT activity in purslane was higher than that of canola, which may suggest that CAT plays a more important role in mitigating the stress imposed by extracts of *Rhus typhina* L. than in canola. There was a single-peak trend with minor fluctuations for CAT activity during purslane germination, whereas a double-peak pattern was observed in canola over the course of germination (Figure 4a–d).

At 5 h of germination for purslane, the CAT activity under treatments with pericarp and fallen leaf at two concentrations was significantly higher than in the control (Figure 4a,b). Specifically, the highest CAT activity in purslane was observed under pericarp (516.63 and 587.12 U/g) after 5 h of imbibition, while its values using the fallen leaf at both concentrations were 450.96 U/g and 428.57 U/g, respectively (Figure 4b).

In canola, the general CAT activity increased to the initial peak after 5 h of imbibition and then decreased within 6–9 h after imbibition. The second peak was detected after 10 h of imbibition under both concentrations of treatments. Across all three extract treatments at both concentrations, the general CAT activity under the three treatments was significantly higher than in the controls (Figure 4c,d) which further confirmed that earlier CAT activity contributes less prominently to antioxidant defense in canola under the stress of extracts of *Rhus typhina* L. than it does in purslane.

### 2.5. Anatomical Structures During Germination of Portulaca oleracea *L.* and Brassica napus *L.* Using Paraffin Sectioning

**Figure 5:**
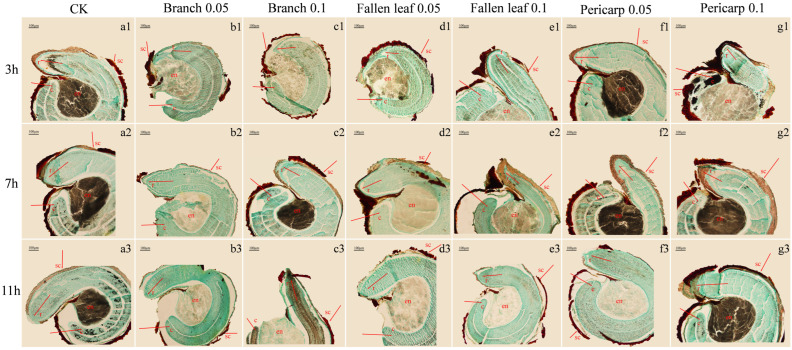

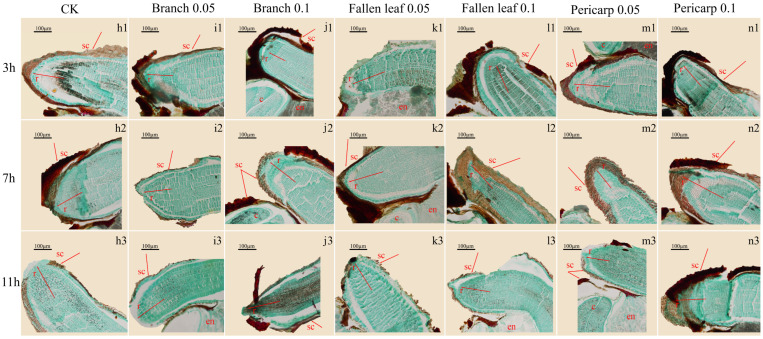
Anatomical structures during germination of *Portulaca oleracea* L. treated with powder of *Rhus typhina* L. at concentrations of 0.05 g/mL and 0.1 g/mL using paraffin sectioning at 3, 7, and 11 h after imbibition under 10× (above) and 20× (below). Note: sc = seed coat; r = radicle; en = endosperm; c = cotyledon. (**a1**) *Portulaca oleracea* L. treated with water as a control after 3 h of imbibition under 10×; (**a2**) *Portulaca oleracea* L. treated with water as a control after 7 h of imbibition under 10×; (**a3**) *Portulaca oleracea* L. treated with water as a control after 11 h of imbibition under 10×. (**b1**) *Portulaca oleracea* L. treated with branch powder at a concentration of 0.05 g/mL after 3 h of imbibition under 10×; (**b2**) *Portulaca oleracea* L. treated with branch powder at a concentration of 0.05 g/mL after 7 h of imbibition under 10×; (**b3**) *Portulaca oleracea* L. treated with branch powder at a concentration of 0.05 g/mL after 11 h of imbibition under 10×; (**c1**) *Portulaca oleracea* L. treated with branch powder at a concentration of 0.1 g/mL after 3 h of imbibition under 10×; (**c2**) *Portulaca oleracea* L. treated with branch powder at a concentration of 0.1 g/mL after 7 h of imbibition under 10×; (**c3**) *Portulaca oleracea* L. treated with branch power at a concentration of 0.1 g/mL after 11 h of imbibition under 10×. (**d1**) *Portulaca oleracea* L. treated with fallen leaf powder at a concentration of 0.05 g/mL after 3 h of imbibition under 10×; (**d2**) *Portulaca oleracea* L. treated with fallen leaf powder at a concentration of 0.05 g/mL after 7 h of imbibition under 10×; (**d3**) *Portulaca oleracea* L. treated with fallen leaf powder at a concentration of 0.05 g/mL after 11 h of imbibition under 10×; (**e1**) *Portulaca oleracea* L. treated with fallen leaf powder at a concentration of 0.1 g/mL after 3 h of imbibition under 10×; (**e2**) *Portulaca oleracea* L. treated with fallen leaf powder at a concentration of 0.1 g/mL after 7 h of imbibition under 10×; (**e3**) *Portulaca oleracea* L. treated with fallen leaf powder at a concentration of 0.1g/mL after 11 h of imbibition under 10×. (**f1**) *Portulaca oleracea* L. treated with pericarp powder at a concentration of 0.05 g/mL after 3 h of imbibition under 10×; (**f2**) *Portulaca oleracea* L. treated with pericarp powder at a concentration of 0.05 g/mL after 7 h of imbibition under 10×; (**f3**) *Portulaca oleracea* L. treated with pericarp powder at a concentration of 0.05 g/mL after 11 h of imbibition under 10×; (**g1**) *Portulaca oleracea* L. treated with pericarp powder at a concentration of 0.1 g/mL after 3 h of imbibition under 10×; (**g2**) *Portulaca oleracea* L. treated with pericarp powder at a concentration of 0.1 g/mL after 7 h of imbibition under 10×; (**g3**) *Portulaca oleracea* L. treated with pericarp powder at a concentration of 0.1g/mL after 11 h of imbibition under 10×. (**h1**) *Portulaca oleracea* L. treated with water as a control after 3 h of imbibition under 20×; (**h2**) *Portulaca oleracea* L. treated with water as a control after 7 h of imbibition 20×; (**h3**) *Portulaca oleracea* L. treated with water as a control after 11 h of imbibition 20×. (**i1**) *Portulaca oleracea* L. treated with branch powder at a concentration of 0.05 g/mL after 3 h of imbibition 20×; (**i2**) *Portulaca oleracea* L. treated with branch powder at a concentration of 0.05 g/mL after 7 h of imbibition 20×; (**i3**) *Portulaca oleracea* L. treated with branch powder at a concentration of 0.05 g/mL after 11 h of imbibition 20×; (**j1**) *Portulaca oleracea* L. treated with branch powder at a concentration of 0.1 g/mL after 3 h of imbibition 20×; (**j2**) *Portulaca oleracea* L. treated with branch powder at a concentration of 0.1 g/mL after 7 h of imbibition 20×; (**j3**) *Portulaca oleracea* L. treated with branch power at a concentration of 0.1 g/mL after 11 h of imbibition 20×. (**k1**) *Portulaca oleracea* L. treated with fallen leaf powder at a concentration of 0.05 g/mL after 3 h of imbibition 20×; (**k2**) *Portulaca oleracea* L. treated with fallen leaf powder at a concentration of 0.05 g/mL after 7 h of imbibition 20×; (**k3**) *Portulaca oleracea* L. treated with fallen leaf powder at a concentration of 0.05 g/mL after 11 h of imbibition 20×; (**l1**) *Portulaca oleracea* L. treated with fallen leaf powder at a concentration of 0.1 g/mL after 3 h of imbibition 20×; (**l2**) *Portulaca oleracea* L. treated with fallen leaf powder at a concentration of 0.1 g/mL after 7 h of imbibition 20×; (**l3**) *Portulaca oleracea* L. treated with fallen leaf powder at a concentration of 0.1g/mL after 11 h of imbibition 20×. (**m1**) *Portulaca oleracea* L. treated with pericarp powder at a concentration of 0.05 g/mL after 3 h of imbibition 20×; (**m2**) *Portulaca oleracea* L. treated with pericarp powder at a concentration of 0.05 g/mL after 7 h of imbibition 20×; (**m3**) *Portulaca oleracea* L. treated with pericarp powder at a concentration of 0.05 g/mL after 11 h of imbibition 20×; (**n1**) *Portulaca oleracea* L. treated with pericarp powder at a concentration of 0.1 g/mL after 3 h of imbibition 20×; (**n2**) *Portulaca oleracea* L. treated with pericarp powder at a concentration of 0.1 g/mL after 7 h of imbibition 20×; (**n3**) *Portulaca oleracea* L. treated with pericarp powder at a concentration of 0.1g/mL after 11 h of imbibition 20×.

**Figure 6:**
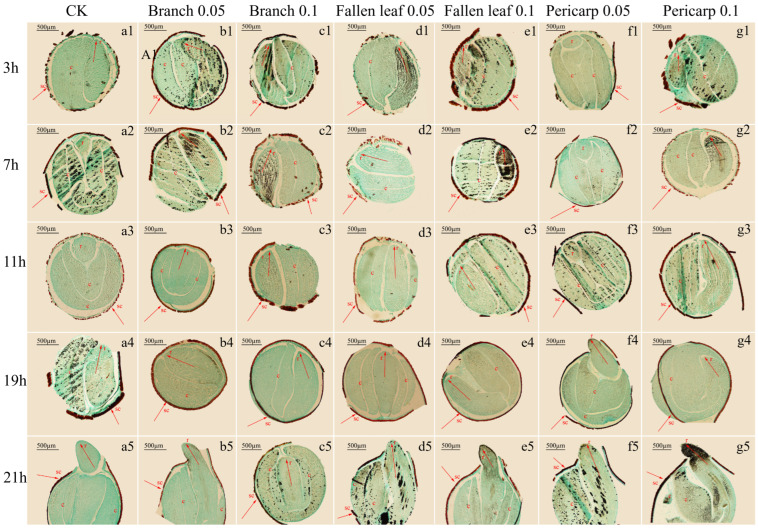

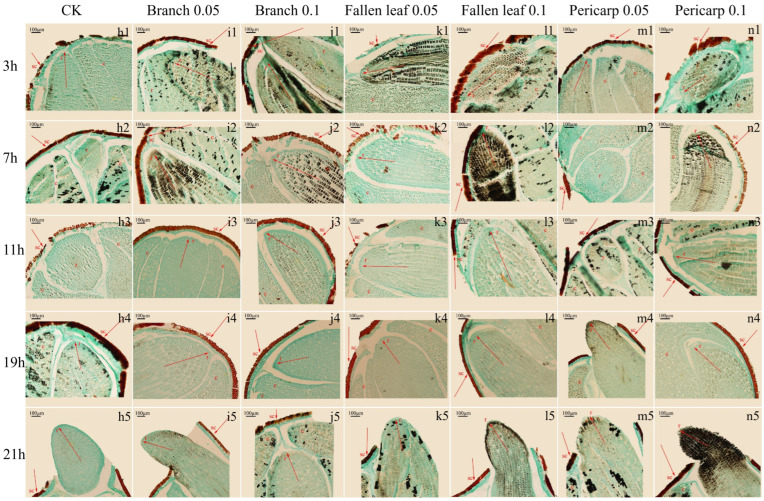
Anatomical structures during germination of *Brassica napus* L. treated with powder of *Rhus typhina* L. at concentrations of 0.05 g/mL and 0.1 g/mL using paraffin sectioning at 3, 7, 11, 19, and 21 h after imbibition under 10× (above) and 20× (below). Note: sc = seed coat; r = radicle; c = cotyledon. (**a1**) *Brassica napus* L. treated with water as a control after 3 h of imbibition under 10×; (**a2**) *Brassica napus* L. treated with water as a control after 7 h of imbibition under 10×; (**a3**) *Brassica napus* L. treated with water as a control after 11 h of imbibition under 10×; (**a4**) *Brassica napus* L. treated with water as a control after 19 h of imbibition under 10×; (**a5**) *Brassica napus* L. treated with water as a control after 21 h of imbibition under 10×. (**b1**) *Brassica napus* L. treated with branch powder at a concentration of 0.05 g/mL after 3 h of imbibition under 10×; (**b2**) *Brassica napus* L. treated with branch powder at a concentration of 0.05 g/mL after 7 h of imbibition under 10×; (**b3**) *Brassica napus* L. treated with branch powder at a concentration of 0.05 g/mL after 11 h of imbibition under 10×; (**b4**) *Brassica napus* L. treated with branch powder at a concentration of 0.05 g/mL after 19 h of imbibition under 10×; (**b5**) *Brassica napus* L. treated with branch powder at a concentration of 0.05 g/mL after 21 h of imbibition under 10×. (**c1**) *Brassica napus* L. treated with branch powder at a concentration of 0.1 g/mL after 3 h of imbibition under 10×; (**c2**) *Brassica napus* L. treated with branch powder at a concentration of 0.1 g/mL after 7 h of imbibition under 10×; (**c3**) *Brassica napus* L. treated with branch powder at a concentration of 0.1 g/mL after 11 h of imbibition under 10×; (**c4**) *Brassica napus* treated with branch powder at a concentration of 0.1 g/mL after 19 h of imbibition under 10×; (**c5**) *Brassica napus* L. treated with branch powder at a concentration of 0.1 g/mL after 21 h of imbibition under 10×. (**d1**) *Brassica napus* L. treated with fallen leaf powder at a concentration of 0.05 g/mL after 3 h of imbibition under 10×; (**d2**) *Brassica napus* L. treated with fallen leaf powder at a concentration of 0.05 g/mL after 7 h of imbibition under 10×; (**d3**) *Brassica napus* L. treated with fallen leaf powder at a concentration of 0.05 g/mL after 11 h of imbibition under 10×; (**d4**) *Brassica napus* L. treated with fallen leaf powder at a concentration of 0.05 g/mL after 19 h of imbibition under 10×; (**d5**) *Brassica napus* L. treated with fallen leaf powder at a concentration of 0.05 g/mL after 21 h of imbibition under 10×. (**e1**) *Brassica napus* L. treated with fallen leaf powder at a concentration of 0.1 g/mL after 3 h of imbibition under 10×; (**e2**) *Brassica napus* L. treated with fallen leaf powder at a concentration of 0.1 g/mL after 7 h of imbibition under 10×; (**e3**) *Brassica napus* L. treated with fallen leaf powder at a concentration of 0.1 g/mL after 11 h of imbibition under 10×; (**e4**) *Brassica napus* L. treated with fallen leaf powder at a concentration of 0.1 g/mL after 19 h of imbibition under 10×; (**e5**) *Brassica napus* L. treated with fallen leaf powder at a concentration of 0.1 g/mL after 21 h of imbibition under 10×. (**f1**) *Brassica napus* L. treated with pericarp powder at a concentration of 0.05 g/mL after 3 h of imbibition under 10×; (**f2**) *Brassica napus* L. treated with pericarp powder at a concentration of 0.05 g/mL after 7 h of imbibition under 10×; (**f3**) *Brassica napus* L. treated with pericarp powder at a concentration of 0.05 g/mL after 11 h of imbibition under 10×; (**f4**) *Brassica napus* L. treated with pericarp powder at a concentration of 0.05 g/mL after 19 h of imbibition under 10×; (**f5**) *Brassica napus* L. treated with pericarp powder at a concentration of 0.05 g/mL after 21 h of imbibition under 10×. (**g1**) *Brassica napus* L. treated with pericarp powder at a concentration of 0.1 g/mL after 3 h of imbibition under 10×; (**g2**) *Brassica napus* L. treated with pericarp powder at a concentration of 0.1 g/mL after 7 h of imbibition under 10×; (**g3**) *Brassica napus* L. treated with pericarp powder at a concentration of 0.1 g/mL after 11 h of imbibition under 10×; (**g4**) *Brassica napus* L. treated with pericarp powder at a concentration of 0.1 g/mL after 19 h of imbibition under 10×; (**g5**) *Brassica napus* L. treated with pericarp powder at a concentration of 0.1 g/mL after 21 h of imbibition under 10×. (**h1**) *Brassica napus* L. treated with water as a control after 3 h of imbibition under 20×; (**h2**) *Brassica napus* L. treated with water as a control after 7 h of imbibition under 20×; (**h3**) *Brassica napus* L. treated with water as a control after 11 h of imbibition under 20×; (**h4**) *Brassica napus* L. treated with water as a control after 19 h of imbibition under 20×; (**h5**) *Brassica napus* L. treated with water as a control after 21 h of imbibition under 20×. (**i1**) *Brassica napus* L. treated with branch powder at a concentration of 0.05 g/mL after 3 h of imbibition under 20×; (**i2**) *Brassica napus* L. treated with branch powder at a concentration of 0.05 g/mL after 7 h of imbibition under 20×; (**i3**) *Brassica napus* L. treated with branch powder at a concentration of 0.05 g/mL after 11 h of imbibition under 20×; (**i4**) *Brassica napus* L. treated with branch powder at a concentration of 0.05 g/mL after 19 h of imbibition under 20×; (**i5**) *Brassica napus* L. treated with branch powder at a concentration of 0.05 g/mL after 21 h of imbibition under 20×. (**j1**) *Brassica napus* L. treated with branch powder at a concentration of 0.1 g/mL after 3 h of imbibition under 20×; (**j2**) *Brassica napus* L. treated with branch powder at a concentration of 0.1 g/mL after 7 h of imbibition under 20×; (**j3**) *Brassica napus* L. treated with branch powder at a concentration of 0.1 g/mL after 11 h of imbibition under 20×; (**j4**) *Brassica napus* L. treated with branch powder at a concentration of 0.1 g/mL after 19 h of imbibition under 20×; (**j5**) *Brassica napus* L. treated with branch powder at a concentration of 0.1 g/mL after 21 h of imbibition under 20×. (**k1**) *Brassica napus* L. treated with fallen leaf powder at a concentration of 0.05 g/mL after 3 h of imbibition under 20×; (**k2**) *Brassica napus* L. treated with fallen leaf powder at a concentration of 0.05 g/mL after 7 h of imbibition under 20×; (**k3**) *Brassica napus* L.treated with fallen leaf powder at a concentration of 0.05 g/mL after 11 h of imbibition under 20×; (**k4**) *Brassica napus* L. treated with fallen leaf powder at a concentration of 0.05 g/mL after 19 h of imbibition under 20×; (**k5**) *Brassica napus* L. treated with fallen leaf powder at a concentration of 0.05 g/mL after 21 h of imbibition under 20×. (**l1**) *Brassica napus* L. treated with fallen leaf powder at a concentration of 0.1 g/mL after 3 h of imbibition under 20×; (**l2**) *Brassica napus* L. treated with fallen leaf powder at a concentration of 0.1 g/mL after 7 h of imbibition under 20×; (**l3**) *Brassica napus* L. treated with fallen leaf powder at a concentration of 0.1 g/mL after 11 h of imbibition under 20×; (**l4**) *Brassica napus* L. treated with fallen leaf powder at a concentration of 0.1 g/mL after 19 h of imbibition under 20×; (**l5**) *Brassica napus* L. treated with fallen leaf powder at a concentration of 0.1 g/mL after 21 h of imbibition under 20×. (**m1**) *Brassica napus* L. treated with pericarp powder at a concentration of 0.05 g/mL after 3 h of imbibition under 20×; (**m2**) *Brassica napus* L. treated with pericarp powder at a concentration of 0.05 g/mL after 7 h of imbibition under 20×; (**m3**) *Brassica napus* L. treated with pericarp powder at a concentration of 0.05 g/mL after 11 h of imbibition under 20×; (**m4**) *Brassica napus* L. treated with pericarp powder at a concentration of 0.05 g/mL after 19 h of imbibition under 20×; (**m5**) *Brassica napus* L. treated with pericarp powder at a concentration of 0.05 g/mL after 21 h of imbibition under 20×. (**n1**) *Brassica napus* L. treated with pericarp powder at a concentration of 0.1 g/mL after 3 h of imbibition under 20×; (**n2**) *Brassica napus* L. treated with pericarp powder at a concentration of 0.1 g/mL after 7 h of imbibition under 20×; (**n3**) *Brassica napus* L. treated with pericarp powder at a concentration of 0.1 g/mL after 11 h of imbibition under 20×; (**n4**) *Brassica napus* L. treated with pericarp powder at a concentration of 0.1 g/mL after 19 h of imbibition under 20×; (**n5**) *Brassica napus* L. treated with pericarp powder at a concentration of 0.1 g/mL after 21 h of imbibition under 20×.

Based on the seed germination dynamics for purslane and canola, the total seed germination time for purslane was 11 h, while it was 21 h for canola (Figure 5 and Figure 6). The first 3 h represented the seed imbibition period for purslane and 4–7 h represented the metabolic activation period (Figure 5(a1–a3)), while subsequent radicle emergence and full germination process occurred between 8 and 12 h without any treatment (Figure 5(a1–a3)). Therefore, in this study, at these three time points, the seed samples were collected for anatomical structure analysis. All treatments under both concentrations inhibited the germination of purslane (Figure 5(b1–g3)). At the end of the initial germination stage, endosperm cells exhibited swelling, deformation, and unclear boundaries between cells. Starch granules were scattered chaotically with deformed and ruptured cell structures (Figure 5(b1–g1)). In contrast, untreated control seeds exhibited clear morphological differentiation, evenly distributed cellular components, and distinct internal structural development with the radicle fully elongating and penetrating the seed coat (Figure 5(a1–a3)).

Under a low (0.05 g/mL) concentration of branch treatment, starch granules were sparsely scattered, while the radicle elongated and broke the seed coat at the later stage of germination (Figure 5(b1–b3),(i1–i3)). However, seeds treated with a high (0.1 g/mL) concentration of branch treatment showed swelling in the endosperm structure and darker coloration, and the radicle failed to penetrate the seed coat by the end of the germination period (Figure 5(c1–c3),(j1–j3)). Under both concentrations of fallen leaf treatments, a very light color of the endosperm was detected, while the radicle did not break the seed coat even at the end of its germination (Figure 5(d1–e3),(k1–l3)). Under low concentrations of pericarp treatment, the staining of the endosperm was dark at the early stage of germination and the radicle did not break the seed coat at the end of its germination with a lighter staining of the endosperm (Figure 5(f1–f3),(m1–m3)). When treated with a high concentration of pericarp, endosperm staining intensified while the radicle did not break the seed coat at the end of its germination (Figure 5(g1–g3),(n1–n3)).

### 2.6. Anatomical Structures During Germination of Brassica napus *L.* Using Paraffin Sectioning

After 3 h of imbibition under the control, the internal structure of the canola seed was intact with a well-organized cell arrangement. Starch grains were distributed within the cotyledons and appeared normal in morphology with a large amount of starch accumulated and appearing dark in cotyledon cells (Figure 6(a1)). Between 7 and 21 h of imbibition, canola seeds exposed to the extract treatments exhibited cell swelling, deformation, and blurred cell boundaries (Figure 6(a1–a5),(h1–h5)). At 21 h of imbibition, the radicle broke through the seed coat successfully and continued normal seedling growth (Figure 6(a5,h5)).

During the early stage of germination for canola without any treatment (3–7 h), large amounts of starch and lipids accumulated in the radicle for promoting radicle elongation (Figure 6(a1,a2),(h1,h2)). In contrast, seeds treated with both concentrations exhibited large disordered black clumps (Figure 6(b1–g2),(i1–n2)). During the later stage of germination (11–19 h), stained reserves of starch and lipids disappeared under treatments of the branch at both concentrations and the fallen leaf at 0.05 g/mL (Figure 6(b3–d4),(i3–k4)). By 21 h, the radicle broke the seed coat to complete successful germination under all treatments at both concentrations except for treatment of the branch at 0.1 g/mL (Figure 6(b5–g5)). However, large amounts of dark stained materials accumulated under both concentrations of the fallen leaf and pericarp at the tip of the radicle, which led to abnormal germination (Figure 6(c5–g5),(j5–n5)).

### 2.7. α-Amylase Activity During Germination of Portulaca oleracea *L.* and Brassica napus *L.*

**Figure 7:**
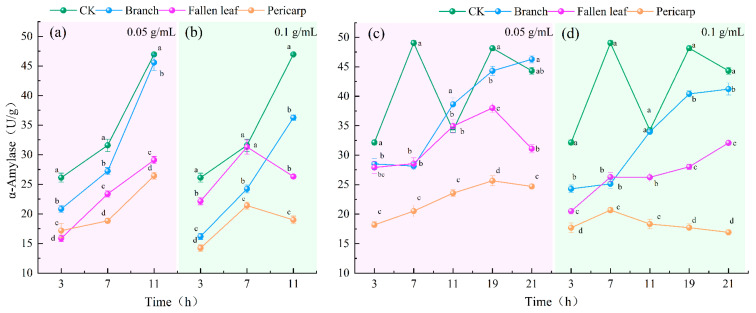
α-Amylase activity during seed germination of *Portulaca oleracea* L. treated with powder of *Rhus typhina* L. at concentrations of 0.05 g/mL (**a**) and 0.1 g/mL (**b**) and *Brassica napus* L. treated with powder of *Rhus typhina* L. at concentrations of 0.05 g/mL (**c**) and 0.1 g/mL (**d**). Different letters above each data point indicate significant differences among different treatments at each time point at α ≤ 0.05.

According to the anatomical structure, three time points (3, 7, and 11 h) were used to evaluate the activity of α-amylase during the seed germination of purslane treated with powder extract of *Rhus typhina* L. In general, the α-amylase activity increased with the time of germination under all treatments and the control (Figure 7a,b). However, the general α-amylase activities under all treatments at both concentrations were significantly lower than the control for all time points. Specifically, at the peak point, the α-amylase activity of the control (49.96 U/g) was significantly higher than that of the 0.05 g/mL branch (45.59 U/g), 0.1 g/mL branch (36.26 U/g), 0.05 g/mL fallen leaf (29.09 U/g), and 0.05 g/mL pericarp (26.43 U/g), respectively. Under 0.1 g/mL of fallen leaf and pericarp, the α-amylase activity decreased to 26.31 and 18.98 U/g, respectively (Figure 7b), which indicated the significant inhibitory effect of *Rhus typhina* L. powder extract against purslane germination.

α-Amylase activity in canola showed a similar trend with similar activities to those of purslane. Under both concentrations of treatments, the α-amylase activity under the pericarp was the lowest compared with the rest of the treatments at all time points (Figure 7c,d). At the end of germination (21 h), under 0.05 g/mL and 0.1 g/mL branch and 0.1 g/mL fallen leaf treatment, the α-amylase activities were 46.28, 41.22, and 32.08 U/g, respectively, while the activities under 0.05 g/mL fallen leaf, 0.05 g/mL pericarp, and 0.1 g/mL pericarp decreased to 31.09, 24.69, and 16.91 U/g, respectively (Figure 7d), which further confirmed the inhibitory effect of *Rhus typhina* L. powder extract against seed germination while such an effect was lower compared with purslane.

### 2.8. β-Amylase Activity During Germination of Portulaca oleracea *L.* and Brassica napus *L.*

**Figure 8:**
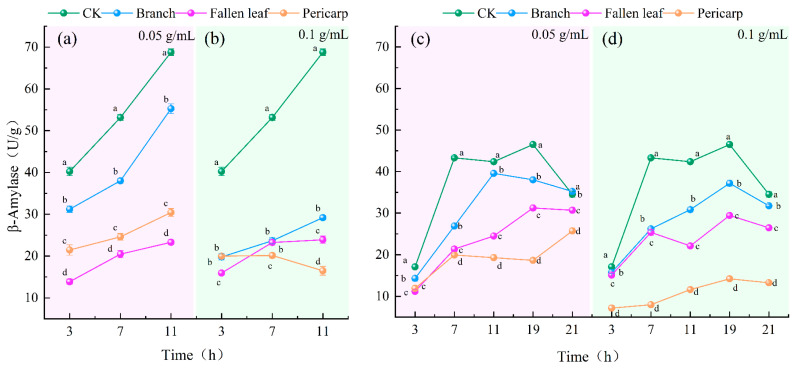
β-amylase activity during seed germination of *Portulaca oleracea* L. treated with powder of *Rhus typhina* L. at concentrations of 0.05 g/mL (**a**) and 0.1 g/mL (**b**) and *Brassica napus* L. treated with powder of *Rhus typhina* L. at concentrations of 0.05 g/mL (**c**) and 0.1 g/mL (**d**). Different letters above each data point indicate significant differences among different treatments at each time point at α ≤ 0.05.

After imbibition, the average β-amylase activities in purslane and canola were similar, while the highest β-amylase activity in purslane (68.78 U/g) under the control at 11 h after imbibition was significantly higher than in canola (46.24 U/g) in general (Figure 8b,d). Its dynamic activities increased before purslane germinated, while two peak points were detected for canola before its germination (Figure 8). Under both concentrations of all treatments, the average β-amylase activity under the control was significantly higher than in all treatments (Figure 8a,b). Specifically, the β-amylase activity under treatment with pericarp (0.1 g/mL) was 16.48 U/g, which was significantly lower than all other treatments (Figure 8b).

In canola, two peak points at 7 and 19 h after imbibition under both concentrations were detected for β-amylase activity, and its activities under the control were significantly higher than in all other treatments (Figure 8c,d). Particularly, under treatment with pericarp at 0.1 g/mL, the β-amylase activity was the lowest (13.25 U/g) at 21 h after imbibition (Figure 8d). The three treatments under the same concentration showed inhibition of β-amylase activity which ranked as branch < fallen leaf < pericarp, confirming the inhibitory effect of *Rhus typhina* L. powder extract against seed germination by inhibiting β-amylase activity.

### 2.9. Correlation Analysis Between Antioxidative Enzyme and Amylase Activities in Portulaca oleracea *L.* and Brassica napus *L.*

**Figure 9:**
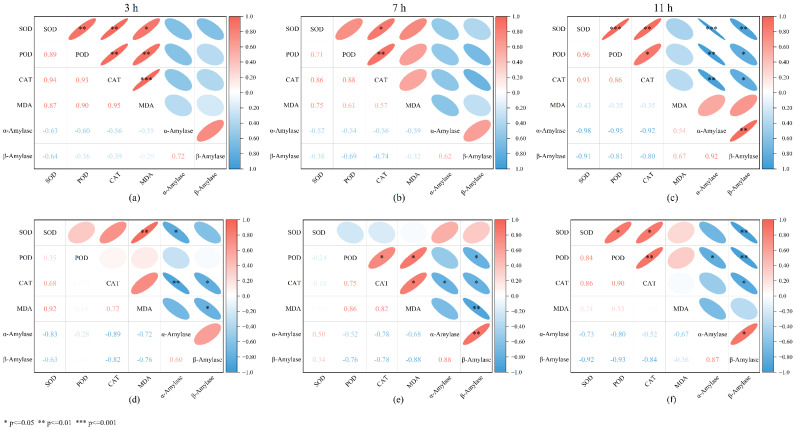
Correlation analysis between antioxidative enzyme and amylase activities during seed germination of *Portulaca oleracea* L. treated with powder of *Rhus typhina* L. at 3 (**a**), 7 (**b**), and 11 h (**c**) and of *Brassica napus* L. at 3 (**d**), 7 (**e**), and 11 h (**f**). The color scale indicates the range of correlation coefficients; orange indicates a positive correlation and blue indicates a negative correlation.

Pearson correlation analyses among SOD, POD, CAT, MDA, α-amylase, and β-amylase at three time points (3 h, 7 h, and 11 h) were conducted in purslane (Figure 9). Three hours after treatment, all antioxidative enzymes of SOD (r = 0.87, *p* < 0.05), POD (r = 0.9, *p* < 0.01), CAT (r = 0.95, *p* < 0.001) showed a highly significant positive correlation with one another. Meanwhile, a marginal positive correlation was observed between α-amylase and β-amylase (r = 0.72). After 7 h of germination for *Portulaca oleracea* L., negative correlations between β-amylase and POD and CAT were detected (r = −0.69 and −0.741; Figure 9b). At 11 h, α- and β-amylase exhibited a highly significant positive correlation (r = 0.92, *p* < 0.01; Figure 9c).

After 3 h of germination for canola, SOD and CAT were significantly positively correlated with MDA (r = 0.92 and 0.72), while POD and MDA were almost uncorrelated (r = 0.14; Figure 9d). After 7 h, there was a negative correlation of MDA with SOD, while significant positive correlations with both POD and CAT (r = 0.86 and 0.82, *p* < 0.05) were found (Figure 9e). After 11 h, significant positive correlations between SOD and POD (r = 0.84; *p* < 0.05), SOD and CAT (r = 0.8; *p* < 0.05), and POD and CAT (r = 0.9; *p* < 0.05; Figure 9f) were also observed.

### 2.10. Principal Component Analysis of Antioxidative Enzyme and Amylase Activities in Portulaca oleracea *L.* and Brassica napus *L.*

**Figure 10:**
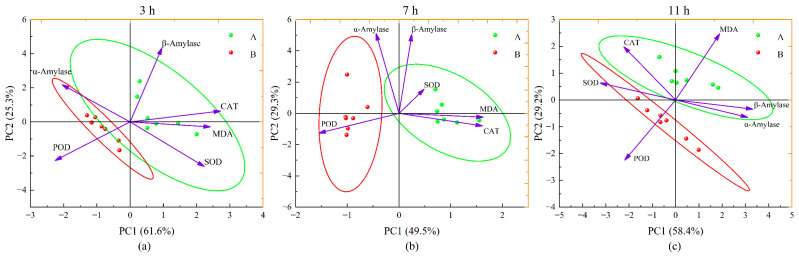
Principal component analysis of major components of antioxidative enzymes and amylase activities in germination of *Portulaca oleracea* L. and *Brassica napus* L. treated with powder of *Rhus typhina* L. at 3 (**a**), 7 (**b**), and 11 h (**c**). A with green circle represents purslane and B with red circle represents canola.

Principal component analysis (PCA) was used to elucidate the intrinsic relationships among antioxidative enzyme activities and overall responses during the germination of purslane and canola under different powder extracts from *Rhus typhina* L. at three time points (3 h, 7 h, and 11 h). The PCA showed that purslane and *canola* were clearly separated along the first or second principal component at all three time points consistently (Figure 10a–c).

The loading vectors of α-amylase and β-amylase consistently point in similar directions and form small angles. The loadings of MDA, SOD, and CAT were co-directional at 3 and 7 h after imbibition for both species, while they appeared to have different directions at 11 h (Figure 10a–c).

### 2.11. Allelopathic Chemical Constitues Determiantion in Rhus typhina *L.*

#### 2.11.1. Standard Curve Preparation for Chemical Compounds Extracted from *Rhus typhina* L. Using LC-MS

Standard compounds were used to quantify the content of chemical compounds in *Rhus typhina* L. using liquid chromatography–mass spectrometry (LC-MS). The mixed standard solution was measured and standard curves were constructed by plotting the mass concentration of all compounds on the *x*-axis against the corresponding peak area on the *y*-axis. All derived linear coefficients of determiantion R^2^ exceeded 0.99, which indicated excellent linearity across the full calibrated concentration range for standard compounds (Table 1).

#### 2.11.2. Content Determination of Compounds in *Rhus typhina* L.

A total of 16 allelochemicals were identified in the branch, fallen leaf, and pericarp of *Rhus typhina* L. including caffeic acid, salicylic acid, methyl 3,4-dihydroxybenzoate, vanillin, gallic acid, methyl gallate, oropol, soy isoflavones, phloretin, kaempferol, naringenin, apigenin, fustin, rutin, quercetin, and tyrosol (Table 2). These 16 compounds in *Rhus typhina* L. could be divided into two categories where the first category was phenolic acids and their derivatives, including caffeic acid, salicylic acid, methyl 3,4-dihydroxybenzoate, gallic acid, methyl gallate and vanillin. The second category was flavonoids including apigenin, daidzein, fustin, naringenin, phloretin, kaempferol, rutin, quercetin, tyrosol and oropol.

In the analyses, 70% methanol and 70% acetonitrile were selected as extraction solvents to compare the recovering targeted compounds from different tissues of *Rhus typhina* L. It was found that the measured content of individual analytes varied with different extraction solvents. For branch tissue, the contents of naringenin and fustin were relatively high when using 70% acetonitrile. In the fallen leaf samples, the content of apigenin, gallic acid, quercetin salicylic acid, phloretin, and vanillin were relatively high when using 70% acetonitrile. For vanillin, 70% methanol was more effective, while for apigenin, gallic acid, quercetin, salicylic acid, and phloretin, 70% acetonitrile was more suitable. For pericarp tissue, methyl dihydroxybenzoate extraction with 70% methanol was more effective than with 70% acetonitrile, whereas for the remaining compounds, the opposite effect was found (Table 2).

### 2.12. Evaluation of Bioherbicidal Activity from Selected Compounds Against Portulaca oleracea *L.*

**Figure 11:**
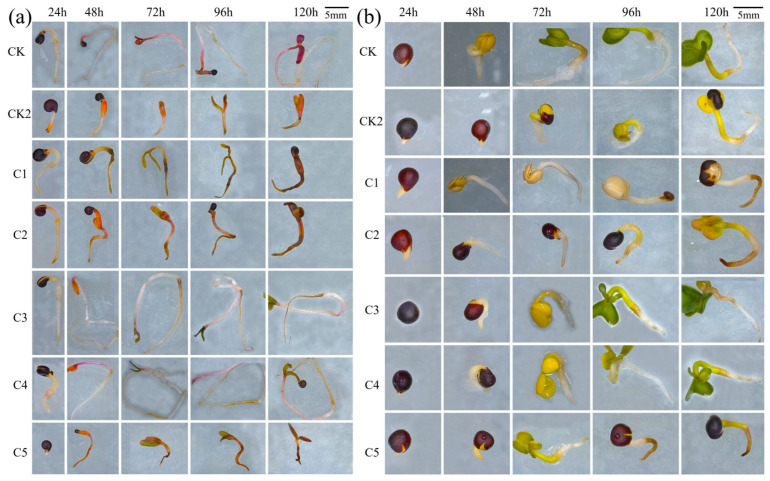
Visual effects of five compounds against seed germination of *Portulaca oleracea* L. (**a**) and *Brassica napus* L. (**b**). Note: CK1 is distilled water; CK2 is herbicide (0.005 g/mL acetochlor); C1 is gallic acid; C2 is methyl gallate; C3 is rutin; C4 is quercetin; C5 is tyrosol.

Based on preliminary screening assays of all compounds previously identified from *Rhus typhina* L., five were confirmed to exhibit a significant inhibitory effect against the seed germination of purslane. Therefore, these five compounds including gallic acid, methyl gallate, rutin, quercetin, and tyrosol at concentrations of 0.02 mg/mL were subsequently selected to further characterize their suppressive effect against the seed germination of purslane. Across the entire germination process, purslane seeds exposed to gallic acid, methyl gallate, and tyrosol developed distinct brown pigmentation and produced markedly shorter radicle length when compared with the solvent-only negative control (CK1; Figure 11a C1, C2, and C5). The inhibitory effects from rutin and quercetin were not as significant as the other three, while the root tip demonstrated a brown color starting from 96 h of treatment (Figure 11a C3,C4). For canola, gallic acid, methyl gallate, and tyrosol exhibited inhibitory effects, while rutin and quercetin did not significantly affect its germination and radicle growth (Figure 11b), which confirmed the earlier observation that *Rhus typhina* L. extracts cause substantially less physiological damage to canola than to purslane.

**Figure 12:**
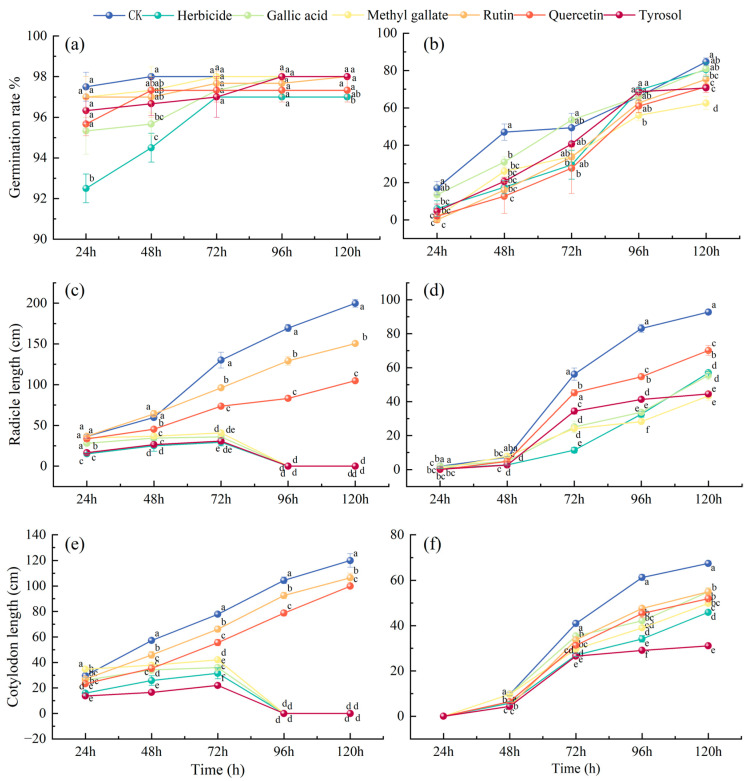
Effects of five compounds against germination rates (**a**,**b**), radicle length (**c**,**d**), and hypocotyl length (**e**,**f**) in *Portulaca oleracea* L. (**a**,**c**,**e**) and *Brassica napus* L. (**b**,**d**,**f**). Different letters above each data point indicate significant differences among different treatments at each time point at α ≤ 0.05.

Qualitative data from seed germination experiments under treatments with all five compounds showed that the germination rate of purslane ranged between 97.33% and 98% after 72–96 h with no significant difference compared to the distilled water (98%) and herbicide (97%) controls (Figure 12a). In contrast, the germination rate of canola after 120 h of treatment with methyl gallate, quercetin, and tyrosol ranged between 62.5% and 75.33% which was significantly lower than that of the distilled water control (84.67%, Figure 12b). Further assessment showed that its radicle length and hypocotyl elongation were all significantly inhibited under treatment with these compounds. After 72 h of treatment, their values decreased to 0 for purslane (Figure 12c,e) while a non-significant effect was identified for canola over the same time course (Figure 12d,f).

After 120 h exposure of rutin and quercetin, the radicle length of purslane (150.46 and 104.84 cm) decreased by 24.7% and 47.5%, respectively, compared to the distilled water control (199.73 cm; Figure 12c,e). For canola, the decrease amount was only 24.4% compared to the distilled water control (92.73 cm; Figure 12d,f).

The purslane hypocotyl length decreased by 13.51 and 20.13 cm (Figure 12c,e), respectively, when compared to the distilled water control (119.97 cm), whereas the hypocotyl length of canola increased slightly by 9.10 and 6.06 cm, respectively (Figure 12d,f), compared to the herbicide control (49.85 cm) under treatment with rutin and quercetin.

## 3. Discussion

### 3.1. Usage of Allelochemicals from Invasive Species Could Provide Green Way of Weed Management

The release of allelochemicals is one of the contributing factors to the success of invasive plants in their non-native ranges [19]. In a New Zealand system, two invasive plants of European origin, Scotch broom (*Cytisus scoparius* (L.) Link) and heather (*Calluna vulgaris* (L.) Hull), co-occur with native species like red tussock (*Chionochloa rubra* Zotov) and mānuka (*Leptospermum scoparium* J.R.Forst. & G.Forst.). 4-O-methylmannose is identified as the dominant compound in broom extract and (E)-pinocarveol in heather extract, while 16-kaurene and methyl palmitate were abundant in both mānuka and tussock extracts, showing a significant effect of invasive plant (heather and broom) root extracts on mānuka germination at all concentrations tested. In this system, adverse effects against seedling growth and biomass at higher concentrations are observed (≥5%) and native species are likely to be more vulnerable to the allelopathic effects of these species [36]. Additionally, regarding invasive species of *Senecio angulatus* L.f., the inhibitory effect of aqueous extracts from leaves and stems could inhibit the early growth of native plants; this effect was stronger than that from belowground parts with higher concentrations and its main potent natural substances are phenols, flavonoids, and antioxidant activities [37]. Therefore, usage of such allelopathic components from invasive plants as biological weapons in green weed management would provide a path for sustainability in agricultural production. *Rhus typhina* L. was introduced from South America and considered as an invasive species. Thus, this study aims to use such an invasive ability to control weeds of *Portulaca oleracea* L.

### 3.2. Effect of Powder from Rhus typhina *L.* Against Antioxidative Enzyme Activities During Germination of Portulaca oleracea *L.*

Allelopathy is a biological activity by which one plant species produces and releases chemical compounds that influence the reproduction, growth, survival, or behavior of other plants via leaching, volatilization, or root exudation of allelochemicals with either beneficial or detrimental effects on the receiver plants [38]. These biochemical compounds can affect critical biological processes such as seed germination, root and shoot elongation, photosynthesis, enzymatic activities, and hormonal balance in neighboring plants [38]. For instance, Mexican sunflower extract significantly suppressed rice germination and growth at 10% of its extraction with abnormal seedling percentage (IC_50_ = 5.59%), reduced radicle length (7.82%), and plumule length (8.81%) [39]. In this study, higher production of MDA due to the pericarp of *Rhus typhina* L. suggests that higher oxidation occurred due to the allelopathic stress caused by the pericarp of *Rhus typhina* L. in purslane seeds. Similarly, with the increase in concentration of fig (*Ficus carica* L.) tree stem aqueous extracts (15.0–25.0 g/L), the content of MDA increased while the activities of SOD and POD of *Siraitia grosvenorii *(Swingle) C.Jeffrey ex A.M.Lu & Zhi Y.Zhang first increased and then decreased [40]. In another study, the IC_50_ from methanolic extracts of leaves of *Synedrella nodiflora* (L.) Gaertn.against mimosa was prolonged and a reduction in insoluble carbohydrates, nucleic acid, and protein contents, as well as amylase activity, was observed [41]. Stress-related antioxidant enzymes of CAT and POD were drastically reduced, while the degree of lipid peroxidation increased [41]. However, in this study, POD activity was higher under this type of treatment which suggests that purslane is able to quench such oxidation (Figure 2). The appearance of two peaks for SOD and CAT indicates that the mechanisms of purslane and canola vary, in which purslane could boost SOD activities twice to dismute the allelopathic stress from powder extract of *Rhus typhina* L. (Figure 3 and Figure 4). The highly positive correlations among antioxidative enzyme activities indicate that oxidative damage and the antioxidant defense system are activated simultaneously during the early stages of stress (Figure 9) which suggests a synergistic relationship between membrane lipid peroxidation and antioxidative enzyme activities.

### 3.3. Effect of Rhus typhina *L. *Against Anatomical Structures During Germination of Portulaca oleracea *L.*

The anatomical structure using paraffin sectioning is one important way of observing anatomical changes during the germination process under different treatments. In this study, untreated seeds of both plant species exhibited a clear and intact internal structure with neatly arranged hypocotyl cells and sparsely distributed starch grains at the early stage (3 h) of germination. The internal *Portulaca oleracea* L. seed cell activity gradually diminished with shorter radicle length and scattered starch granules than the control, which indicates that a high concentration of treatment from *Rhus typhina* L. inhibited the seed germination of purslane by preventing the radicle from breaking through the seed coat and delaying embryo differentiation by slowing down the consumption of starch granules and reducing embryo vitality and radicle elongation (Figure 5).

### 3.4. Effect of Rhus typhina *L.* Against Amylase Activities During Germination of Portulaca oleracea *L.*

The amylase activities in purslane and canola were inhibited due to treatment with powder from *Rhus typhina* L., while this inhibitory effect during the germination of purslane was higher than in canola, which confirmed that the inhibitory effect from the pericarp is the highest compared to all other treatments. It was found that the activity levels of amylase began to rise slowly from 0 to 3 h after seed imbibition in purslane, then increased sharply from 3 to 11 h, reaching a peak which marked the most vigorous starch hydrolysis, with starch being extensively and rapidly degraded (Figure 7). The generated sugars were rapidly utilized for respiration by providing energy for cell division and elongation growth of the radicle and plumule. This confirmed that pericarp at the high concentration showed the highest inhibition of seed germination in purslane due to low α-amylase activity. Compared to β-amylase, α-amylase activity exhibited a brief lag in its rise. After 3 h, the amylase activity under other powder treatments was higher than that under water control treatment, with relatively stable activity changes observed at 7 and 11 h, indicating that under other treatments, amylase maintained a lower activity level, producing a small amount of sugar, and high energy consumption appeared at this time point. The significant positive correlation between α- and β-amylase in purslane exhibited significant fluctuations resulting from allelopathic stress caused by the extract of *Rhus typhina* L. and its inhibition of germination (Figure 9a–c).

### 3.5. Allelochemical Identification in Rhus typhina *L.*

In this study, phenolic acids and their derivatives, including caffeic acid, salicylic acid, methyl 3,4-dihydroxybenzoate, and vanillin and flavonoids including apigenin, daidzein, fustin, naringenin, phloretin, kaempferol, and oropol were identified from the branch, fallen leaf, and pericarp of *Rhus typhina* L. In another study, the major metabolites identified using GC–MS present in the methanolic extracts of *Senecio nodiflora* C.C.Chang responsible for allelopathic activity were quinic acid, protocatechuic acid, gentisic acid, caffeic acid, and ferulic acid, etc. [41]. Additionally, through GC-MS analysis, endoborneol was identified as a key compound in the leachates of *Pinus koraiensis* Siebold & Zucc. which could directly activate defense-related pathways of *Panax ginseng* C.A.Mey. to induce resistance and indirectly recruit its rhizospheric beneficial microbiota by enhancing the secretion of ginsenosides, thereby triggering induced systemic resistance. These results reveal the multifaceted functions of allelochemical endoborneol in disease suppression in agroforestry systems and highlight its potential as an environmentally friendly compound for sustainable agriculture [42]. Therefore, further experiments in this study were conducted to evaluate their inhibitory effects against the seed germination of purslane individually to confirm their effects in weed suppression. Although the germination rate of purslane seeds was not affected, the growth of its seedlings (radicle and hypocotyl) was particularly sensitive to the tested compounds, exhibiting growth arrest and tissue browning under various treatments. The degree of inhibition was generally higher than that observed in canola. In another similar study, compared to the control, the common physical parameters related to shoot and root length and dry and fresh weight were affected in treated seedlings due to the extract of *Synedrella nodiflora* (L.) Gaertn. against mimosa as well [41].

### 3.6. Biosafety Evaluation of Powder from Rhus typhina on Brassica napus *L.*

Canola (*Brassica napus* L.) is a nonendospermic dicotyledonous plant and the energy required for growth is supplied by the cotyledons during germination. Biosafety evaluation of any chemical or biochemical compounds controlling weeds needs to be conducted. PCA results demonstrate that the two plant species exhibit differences in their response to powder extract from *Rhus typhina* L. at different time points. A gradual increase in the germination capacity and enhanced internal tissue activity of canola is also demonstrated by its differentiated starch granule distribution using the paraffin sectioning method (Figure 6).Although allelopathic stress caused an oxidative effect in canola, that effect was significantly lower than in purslane. This may not cause significant harm to canola when using powder extract to suppress purslane in canola fields. Moreover, peroxidation caused by allelopatic stress could be quenched by antioxidative enzymes. For instance, higher POD activity further suggests a higher antioxidative ability in canola than in purslane using *Rhus typhina* L. powder extracts. This conclusion is also confirmed by the appearing of double peaks for CAT activity during canola germination under both concentrations. Inhibition of seed germination in canola under treatments with branch and fallen leaf at both concentrations was lower than with pericarp at two concentrations, which indicates less damage using the powder extract of *Rhus typhina* L.

Correlation analysis further confirms that the defense system for canola was partially activated to defend against the stress from the powder extract of *Rhus typhina* L. The non-significant negative correlation between α- and β-amylase (r = 0.6) at 3 h of germination for canola indicates overall metabolic adjustment to defend against the stress from powder extract of *Rhus typhina* L. These results indicate the possibility of using extracts from *Rhus typhina* L. to inhibit seed germination as a preemergence herbicide in canola fields.

## 4. Materials and Methods

### 4.1. Experimental Material Preparation

The current-year branch, fallen leaf, and fruit head of the *Rhus typhina* L. tree were collected from Xiaoheihe Park in Hohhot, Inner Mongolia, China (40.783901° N, 111.731158° E). *Rhus typhina* L. was identified based on plant fruit, leaf, and photos collected by Dr. Feng Han from the Department of Botany in the College of Grassland Science of Inner Mongolia Agricultural University. Pericarp was peeled from the fruit head manually and air-dried under room temperature (25 °C). Fine powders of the branch, fallen leaf, and pericarp were obtained using a grinder (Shanghai Jingxin Industrial Co., Ltd., Shanghai, China) for further usage. Seeds of purslane (*Portulaca oleracea* L. ) and canola (*Brassica napus* L.) were purchased from the seed industry (Wancaotongyuan Nursery and Floriculture Co., Ltd., Hohhot, China) and stored in a refrigerator at 4 °C for further usage. The seeds of these two plants were grown in pots and botanically identified as *Portulaca oleracea* L. and *Brassica napus* L. at their fruit stage by Dr. Feng Han as well.

### 4.2. Preparation of Agar Medium with Powder of Rhus typhina

In total, 10 g of agar powder was dissolved in 500 mL of distilled water and sterilized in an autoclave (Zhiwei Instruments Co., Ltd., Nanjing, China). After sterilization, 50 g of fine powder from the branch, fallen leaf, and pericarp was added to the agar solution and mixed thoroughly to obtain a 0.1 g·mL^−1^ powder agar stock solution after cooling slightly. A 0.05 g·mL^−1^ solution was prepared by diluting from the stock solution.

### 4.3. Experimental Design

Combinations of two concentrations of 0.05 and 0.1 g/mL of powder from the pericarp, fallen leaf, and branch were used as experimental treatments, respectively (Table 3). Distilled water was used to serve as the control. Before the powdered agar became solid, it was evenly poured into a sterilized flask. Sterilized seeds from purslane and canola were then placed in the flasks with the corresponding treatment combinations. The well-prepared flasks were subsequently transferred to an incubator with a temperature of 25 °C and 40% humidity for seed germination.

### 4.4. Experimental Material Sampling

For malonaldehyde (MDA) content, antioxidative enzyme activity, and amylase activity determination, the seeds of purslane and canola were carefully collected using forceps at 3, 7, and 11 h after they were placed into the agar and preserved in a −80 °C freezer until use. For paraffin sectioning observation, the seeds of purslane were carefully collected using forceps at 3, 7, and 11 h after they were placed into the agar and preserved in FAA solution (V_formaldehyde_: V_glacial acetic acid_: V_50% ethanol_ = 1: 1.2: 17.8), while seeds of canola were carefully collected using forceps at 3, 7, 11, 19, and 21 h after they were placed into the agar and preserved in FAA solution due to their germination dynamics.

### 4.5. Trait Measurement

#### 4.5.1. Malonaldehyde Content

Malonaldehyde (MDA) content was quantified as described by Gao [43]. Frozen seed samples (0.5 g) were homogenized into a fine powder and transferred to a 15 mL centrifuge tube. In total, 10 μL of 10% (w/v) trichloroacetic acid was added and the homogenate and was kept on ice. After centrifugation of the homogenate at 4000× *g* (Sigma-Aldrich, Kappelweg, Burladingen, Germany) for 10 min at 4 °C, the supernatant was collected. A 2 mL aliquot of supernatant was mixed with 2 mL of 0.6% thiobarbituric acid, heated in boiling water for 15 min, and rapidly cooled in an ice bath and centrifuged again (4000× *g*, 10 min). Absorbance of the supernatant was measured at 532 nm and 600 nm in a photospectrometer. MDA content (μmol/g fresh weight) was calculated using the extinction coefficient (155 mM^−1^/cm).

#### 4.5.2. Antioxidative Enzyme Extraction and Assays

Frozen seed samples (1 g) were homogenized in a buffer solution which contains 100 mM Tris-HCl (pH 7.5) with the presence of dithiothreitol (5 mM), EDTA (1.0 mM), MgCl_2_ (10 mM), polyvinyl pyrrolidone (PVP, 1.5%), magnesium acetate (5.0 mM), and aprotinin (1 μg/mL) for enzyme extraction [44].

The activity of superoxide dismutase (SOD) was measured based on the ability of the enzyme to inhibit the light-dependent reduction of nitro blue tetrazolium chloride (NBT). The mixture of enzyme extraction and NBT was measured at 560 nm in a photospectrometer. The amount of enzyme required to produce a 50% inhibition in the photoreduction rate of NBT was defined as one unit of SOD activity calculated as enzyme units per gram of fresh weight [44].

The activity of catalase (CAT) was measured based on the reduction ability of hydrogen peroxide (H_2_O_2_) [33]. The absorbance of the mixture for enzyme extraction (3 mL) and 0.3% H_2_O_2_ (pH 7.0, 50 mmol/L) at 240 nm was recorded for 3 min. Then, 0.3% of H_2_O_2_, 0.2% of guaiacol (50 mmol/L, pH 7.4), and enzyme extract was mixed to form a 3 mL reaction system, and absorbance at 240 nm was recorded for 3 min. Activity was calculated as units per gram of fresh weight.

POD activity was determined using guaiacol oxidation [44]. The 3 mL reaction mixture included enzyme extract, 0.3% H_2_O_2_, 0.2% guaiacol (50 mM, pH 7.4), and 0.1% resorcinol. Absorbance at 470 nm was monitored for 3 min. Activity was calculated as units per gram of fresh weight.

### 4.6. Amylase Activity Assay

#### 4.6.1. α-Amylase Activity Assay

An α-amylase (α-AL) activity assay was performed according to the instructions described in the kit (Beijing Box Biotech Co., Ltd., Beijing, China). Briefly, 0.1 g of frozen sample was mixed with 1 mL of distilled water and extracted at room temperature for 15 min by shaking every 5 min. After extraction, the mixture was centrifuged (6000× *g*, 10 min) at room temperature. The supernatant was collected and diluted to 10 mL with distilled water. Prior to the assay, the microplate reader (Thermo Fisher Scientific China Co., Ltd., Shanghai, China) was preheated for at least 30 min and the instrument was zeroed with distilled water at a wavelength of 540 nm. Then, 2 mL of the amylase stock solution was boiled in a water bath for 5 min and then cooled. The reaction solution was added to the corresponding tubes of test, control, standard, and blank according to the instructions. Finally, 200 μL of each reaction solution was transferred to a 96-well plate and the absorbance (A) of the control, standard, and blank was recorded at 540 nm. A control tube was included for each test, while blank tubes were measured 1~2 times. ΔA_test_ = A_test_ − A_control_ and ΔA_standard_ = A_standard_ − A_blank_ were calculated. A standard curve was constructed using concentrations of 0.2, 0.1, 0.05, 0.025, 0.0125, and 0.00625 g/mL of glucose. The linear regression was obtained as y = 4.0284x + 0.00775. One unit (U) of α-amylase activity was defined as the amount of reducing sugar catalyzed per gram of sample per minute.

$$\alpha -\mathrm{AL}\;\left(U/g\right)=\frac{{X\alpha \times V}_{y}\times Vz}{V_{y}\times W\times T}$$

where V_y_ is the volume of enzyme solution added to the reaction system (0.25 mL), Xα is the absorbance at a wavelength of 540 nm, V_Z_ is the volume of the amylase stock solution (10 mL), W is the sample mass (g), and T is the reaction time (5 min).

#### 4.6.2. β-Amylase Activity Assay

A β-amylase (β-AL) activity assay was performed according to the instructions described in the kit (Beijing Box Biotech Co., Ltd., Beijing, China). Briefly, 0.1 g sample was homogenized with 1 mL of distilled water and extracted at room temperature for 15 min by shaking every 5 min. After extraction, the mixture was centrifuged (6000× *g*, 10 min) at room temperature. The supernatant was collected and diluted to 10 mL with distilled water to obtain the stock solution. An aliquot of the stock solution was boiled in a water bath for 5 min and cooled to obtain inactivated stock solution. A separate aliquot was diluted with 5-fold of distilled water for total (α + β) amylase activity determination. Prior to the assay, the microplate reader (Thermo Fisher Scientific China Co., Ltd., Shanghai, China) was preheated for at least 30 min and the instrument was zeroed with distilled water at a wavelength of 540 nm. Reaction solutions were added to the Test 1, Control 1, Test 2, Control 2, Standard, and Blank tubes. The absorbance of each tube were measured at 540 nm by yielding A1_sample_, A1_control_, A2_sample_, A2_control_, A_standard_ and A_blank_. Calculations of ΔA_α_ = A1_sample_ − A1_control_, ΔA_(α+β)_ = A2_sample_ − A2_control_, and ΔA_standard_ = A_standard_ − A_blank_ were conducted. A standard curve was prepared as described as α-amylase, and ΔA_α_ and ΔA_(α+β)_ were used to obtain X_α_ and X_z_ (mg/mL). One unit (U) of β-amylase activity was defined as the amount of reducing sugar catalyzed per gram of sample per minute.

$$\alpha -\mathrm{AL}\;\left(U/g\right)=\frac{x_{\alpha }\times V_{y}\times Vz}{V_{y}\times W\times T}$$

$$\left(\alpha +\beta \right)-\mathrm{AL}\left(U/g\right)=\frac{5\times x_{z}\times V_{y}\times Vz}{V_{y}\times W\times T}$$

$$\beta -\mathrm{AL}\;\left(U/g\right)=\frac{5\times x_{z}\times V_{y}\times Vz}{V_{y}\times W\times T}-\frac{x_{\alpha }\times V_{y}\times Vz}{V_{y}\times W\times T}$$

where V_y_ is the volume of enzyme solution added to the reaction system (0.25 mL), V_Z_ is the volume of the amylase stock solution (10 mL), W is the sample mass (g), and T is the reaction time (5 min).

### 4.7. Paraffin Sectioning

Conventional paraffin sectioning with modification was employed to examine the cross section structure of seed germination dynamics under treatment with powder from *Rhus typhina* L. All seed sample preservation was carried out using dehydration by the following ethanol gradient: 70% ethanol with one hour, 80% ethanol for an hour, 95% ethanol for an hour, and anhydrous ethanol with one hour two times. Clearing of the samples was performed based on stepwise ethanol and xylene (V/V = 1:1): anhydrous ethanol: xylene (V/V = 1:1) for 1~2 h followed by xylene for 1 h two times. After clearing, the material was transferred to a mixture of xylene and crushed paraffin (V/V = 1:1) and placed in an oven overnight with the temperature gradually increased from 40 to 58 °C. The processed samples were infiltrated with wax for 12~24 h and were kept in pure paraffin for at least 10 h with paraffin every 4 h. After two paraffin changes, samples were embedded, sectioned (10 μm thickness), spread in a 40 °C water bath, and dried at 42 °C. Sections were deparaffinized in xylene, rehydrated through an ethanol gradient, and double-stained with carmine-eosin. Finally, sections were mounted with neutral resin, air-dried in a cool, dark place, and stored permanently. A microscope was used to examine the anatomy structure of these sections from both treated and control treatments. To document the anatomical features, the slides were captured with photographs.

### 4.8. Allelopathic Chemical Profiling in Rhus typhina *L.*

#### 4.8.1. Extraction of Allelopathic Components in *Rhus typhina* L.

In total, 1 g of each powdered sample of pericarp, fallen leaf, and branch from *Rhus typhina* L. was prepared and 20 mL of 70% methanol solution was added to the corresponding extraction tube for extraction under ultrasonic conditions (Xinzhi Biotechnology Co., Ltd., Nanjing, China) for 30 min and then cooled to room temperature. After extraction, samples were shaken well and filtered through qualitative filter paper. The filtrates were condensed to near dryness using a nitrogen evaporator at 50 °C. The condensed residue was dissolved in 2 mL of methanol, filtered through a 0.22 μm organic-phase membrane, and transferred into a sample vial for subsequent analysis. Meanwhile, 70% acetonitrile as the extraction solvent was used to perform the same procedure for extraction of the allelopathic components in *Rhus typhina* L.

#### 4.8.2. Preparation of Standards

In total, 10 mg of each standard chemical was weighed in a balance (Mettler-Toledo, Greifensee, Switzerland) and dissolved in 5 mL methanol to prepare a stock solution at a concentration of 2 mg/mL. A stepwise dilution was conducted for the stock standard solution to 15.62, 31.25, 62.50, 125.00, 250.00, 500.00, and 1000.00 μg/L using methanol. A standard calibration curve was constructed by plotting the mass concentration of each compound (*x*-axis) against the corresponding chromatographic peak area (*y*-axis) yielding the linear equation with a coefficient of determiantion (R^2^).

#### 4.8.3. Chromatographic Conditions

A GOLD C18 solid-phase extraction column (2.1 mm × 100 mm, 1.9 μm; Thermo Fisher Scientific, Shanghai, China) was used as the chromatographic column. The flow rate was 0.2 mL/min, the column temperature was 40 °C and the injection volume was 1 μL. Mobile phase A was ultra-pure water and mobile phase D was acetonitrile. The gradient elution program was prepared and used to determine the allelopathic components in *Rhus typhina* L. (Table 4).

#### 4.8.4. Mass Spectrometry Conditions

The electrospray ionization source of the mass spectrometer was operated in multiple reaction monitoring (MRM) mode (ESI^+^/ESI^−^ mode). The interface voltage of the ion source was set as 2.4 kV. The auxiliary gas flow rate was 580 L/h, the sheath gas flow rate was 165 L/h, and the purge gas flow rate was 240 L/h. The ion transfer tube temperature was 320 °C and the evaporator temperature was 350 °C. The sample solutions were injected and measured automatically. The mass fraction of the target component in the sample (w) was calculated according to the formula.

$$w=\frac{\rho \times v\times d}{m}$$

where w is mass fraction of the compound in the sample (μg/g), ρ is the mass concentration of the compound in the sample solution (μg/mL), v is the constant volume of the sample solution (mL), m is the sample mass (g), and d is the dilution factor.

### 4.9. Evaluation of Bioherbicidal Activity from Selected Compounds Against Portulaca oleracea *L.*

Solutions of 3,4,5-trihydroxybenzoic acid, methyl 3,4,5-trihydroxybenzoate, rutin, tyrosol, and quercetin at concentrations of 0.02 mg/mL were prepared using distilled water. Distilled water (CK1) and commercially available herbicide (CK2) were used as negative and positive controls, respectively.

One hundred seeds of purslane and canola were selected and evenly spread on a Petri dish with two layers of filter paper. In total, 4 mL of the prepared compound solution was added to the Petri dish and placed in an incubator by maintaining at 25 °C and 40% humidity. During the germination process, liquid was replenished in the Petri dishes at fixed time points each day with the volume adjusted to ensure no water film remained on the surface. Each treatment was repeated three times. The number of germinated seeds was recorded and radicle and hypocotyl lengths were measured at the same time. Germination rate was calculated as (%) = (Number of germinated seeds/Total number of test seeds) × 100.

### 4.10. Statistical Analysis

The raw data were organized and preprocessed using Microsoft Excel 2019. Both amylase and antioxidative enzyme activities were subjected to Levene’s test for homogeneity of variances and the Kolmogorov–Smirnov test for normality using SPSS 27 (Amherst, NY, USA). One-way analysis of variance (ANOVA) was evaluated to demonstrate the effects of different treatments on each parameter at the same time point using SPSS 27 (Amherst, NY, USA). Correlation analysis was performed using Pearson’s correlation coefficient to explore correlationships among the parameters. After reducing the dimensionality of the parameter data, principal component analysis (PCA) was applied to calculate composite scores. Various visualizations were generated, including correlation heatmaps and principal component analysis plots using Origin Pro 2024 (Origin Lab, Northampton, MA, USA). All statistical data were presented as the mean ± standard deviation. Statistical significance was indicated at the levels of α ≤ 0.05, while non-significant comparisons were indicated at α > 0.05.

## 5. Conclusions

MDA as a marker of lipid peroxidation and oxidative stress due to treatment with *Rhus typhina* L. extract increased initially and decreased during the later germination stage of *Portulaca oleracea* L., while antioxidative enzymes acted to reduce such oxidative stress with a time lag of MDA. Therefore, the antioxidative enzyme activities were not inhibited due to powder treatments with *Rhus typhina* L. However, the activities of amylase were inhibited by *Rhus typhina* L. powder, which may contribute to the inhibition of seed germination against *Portulaca oleracea* L. as demonstrated by seed structural examination. Phenolic acids and their derivatives, including caffeic acid, salicylic acid, methyl 3,4-dihydroxybenzoate, gallic acid, methyl gallate, and vanillin, and flavonoids including apigenin, daidzein, fustin, naringenin, phloretin, kaempferol, rutin, quercetin, tyrosol, and oropol were the main components identified in the powder of *Rhus typhina* L. Three of these were identified as exhibiting the most significant inhibitory effect against *Portulaca oleracea* L.: gallate, quercetin, and tyrosol. Future studies could be conducted to evaluate the bioherbicidal activity of *Rhus typhina* L. under greenhouse and field conditions, and further molecular mechanisms underlying germination inhibition could be elucidated as well.

## Figures and Tables

**Table 1:** Standard chemical composition of extract from *Rhus typhina* L. using LC-MS.

Compound	Mode	Linear Regression Equation	Coefficient of Determination (R^2^)
Apigenin	ESI^−^	y = 9293x + 93.88	0.9942
	ESI^+^	y = 9531x + 269	0.9972
Salicylic acid	ESI^−^	y = 86,600x + 1184	0.9984
Oropol	ESI^+^	y = 2962x + 18.86	0.9993
Soy isoflavones	ESI^+^	y = 21,530.9x + 261.6	0.9949
Phloretin	ESI^−^	y = 79,590x + 295.7	0.9993
	ESI^+^	y = 12,510x + 118.1	0.998
Kaempferol	ESI^−^	y = 2801x–74.31	0.9979
	ESI^+^	y = 2737x + 53.33	0.9951
Naringenin	ESI^−^	y = 42,340x + 11.71	0.9995
	ESI^+^	y = 13,850x + 242.9	0.9944
Fustin	ESI^+^	y = 2092x–30.68	0.9974
Vanillin	ESI^−^	y = 20,280x + 629.5	0.9946
	ESI^+^	y = 13,800x + 1500	0.9965
Caffeic acid	ESI^+^	y = 44,500x + 754.6	0.9919
Methyl 3,4- dihydroxybenzoate	ESI^+^	y = 64,390x + 270.8	0.9974
Gallic acid	ESI^−^	y = 21,840x	1.0000
Methyl gallate	ESI^+^	y = 523,900x	1.0000
Rutin	ESI^+^	y = 8454x−143.1	0.9982
Quercetin	ESI^−^	y = 5610x−44.44	0.9998
Tyrosol	ESI^−^	y = 351.9x + 132.4	0.9968

**Table 2:** Contents of compounds in different parts of *Rhus typhina* L.

Category	Compound	Mode	Branch (μg/g)		Fallen Leaf (μg/g)		Pericarp (μg/g)	
			70% Methanol	70% Acetonitrile	70% Methanol	70% Acetonitrile	70% Methanol	70% Acetonitrile
Phenolic acids and their derivatives	Caffeic acid	ESI^+^	1.1	0.42	0.78	\	1.22	\
	Salicylic acid	ESI^−^	12.1	17.02	24.78	29.16	19.74	25.12
	Methyl 3,4- dihydroxybenzoate	ESI^+^	3.4	1.52	2.6	0.46	7.02	4.74
	Vanillin	ESI^−^	1.98	8.18	1.4	13.8	2.28	4.82
		ESI^+^	179.2	15.32	425.14	281.7	372.7	67.34
	Gallic acid	ESI^−^	364.92	608.96	4962.12	5356.96	4894.28	4503.58
	Methyl gallate	ESI^+^	46.26	52.58	53.34	58.94	74.18	80.82
Flavonoids	Oropol	ESI^+^	47.58	69.8	36.52	36.02	124.1	157.96
	Daidzein	ESI^+^	0.94	\	\	0.22	0.02	0.38
	Phloretin	ESI^−^	\	\	\	0.36	0.72	0.6
		ESI^+^	3.7	5.72	5.72	8.12	1.06	4.16
	Kaempferol	ESI^−^	3.3	24.22	5.38	14.88	3.72	6.6
		ESI^+^	51.24	75.3	39.28	38.72	134.08	170.72
	Naringenin	ESI^−^	3.3	3.78	0.44	1.24	1.64	1.9
		ESI^+^	3.5	3.68	1.52	2.52	2.46	5.76
	Apigenin	ESI^−^	1.2	2.16	4.28	8.26	2.86	5.02
		ESI^+^	3.84	4.12	22.9	32.98	9.94	17.74
	Fustin	ESI^+^	513.12	1916.76	691.88	421.48	181.06	1629.08
	Rutin	ESI^+^	34	34.54	47.68	58.48	250.12	216.86
	Quercetin	ESI^−^	216.38	272.2	327.7	537.08	250.98	203.7
	Tyrosol	ESI^−^	10.18	9.88	15.68	11.72	5.02	28.82

**Table 3:** Experimental treatments with different concentration of *Rhus typhina* L. powder.

Treatment	CK (Distilled Water)	Pericarp (g/mL)	Fallen Leaf (g/mL)	Branch (g/mL)
1	0	0.05	0.05	0.05
2	-	0.1	0.1	0.1

Note: “-” indicates not applicable.

**Table 4:** Gradient elution procedure for the determination of 16 compounds by liquid chromatography.

Elution Time/min	Volume Fraction%	
	Mobile Phase A (Ultra-Pure Water)	Mobile Phase D (Acetonitrile)
0	90	10
0.1–0.5	75	25
0.51–1.0	60	40
1.1–3.5	10	90
3.50–3.51	90	10
3.51–5.0	90	10

## Data Availability

The original contributions presented in this study are included in the article. Further inquiries can be directed to the corresponding author.
